# A pan-cancer somatic mutation embedding using autoencoders

**DOI:** 10.1186/s12859-019-3298-z

**Published:** 2019-12-11

**Authors:** Martin Palazzo, Pierre Beauseroy, Patricio Yankilevich

**Affiliations:** 1 Instituto de Investigación en Biomedicina de Buenos Aires (IBioBA)—CONICET—Partner Institute of the Max Planck Society, Godoy Cruz 2390, Buenos Aires, C1425FQD Argentina; 2 Institut Charles Delaunay, Universite de Technologie de Troyes, 12 Rue Marie Curie, Troyes, 10300 France; 3 Universidad Tecnologica Nacional, Facultad Regional Buenos Aires, Av. Medrano 951, Buenos Aires, C1179AAQ Argentina

**Keywords:** Autoencoder, Kernel learning, Cancer genomics

## Abstract

**Background:**

Next generation sequencing instruments are providing new opportunities for comprehensive analyses of cancer genomes. The increasing availability of tumor data allows to research the complexity of cancer disease with machine learning methods. The large available repositories of high dimensional tumor samples characterised with germline and somatic mutation data requires advance computational modelling for data interpretation. In this work, we propose to analyze this complex data with neural network learning, a methodology that made impressive advances in image and natural language processing.

**Results:**

Here we present a tumor mutation profile analysis pipeline based on an autoencoder model, which is used to discover better representations of lower dimensionality from large somatic mutation data of 40 different tumor types and subtypes. Kernel learning with hierarchical cluster analysis are used to assess the quality of the learned somatic mutation embedding, on which support vector machine models are used to accurately classify tumor subtypes.

**Conclusions:**

The learned latent space maps the original samples in a much lower dimension while keeping the biological signals from the original tumor samples. This pipeline and the resulting embedding allows an easier exploration of the heterogeneity within and across tumor types and to perform an accurate classification of tumor samples in the pan-cancer somatic mutation landscape.

## Background

Recent years have been characterized by the availability of data repositories providing access to large-scale collaborative cancer projects [1, 2]. These databases contain data from thousands of tumor samples from patients all over the world labeled by tumor type, subtype and other clinical factors such as age and prognosis. The available tumor data includes different layers of biological signals acquired by state-of-the-art omics technologies (e.g., genomics, transcriptomics, proteomics, metabolomics, etc). The information includes somatic mutations, copy number somatic mutations, gene expression, DNA methylation among other data types. Each layer represents the signature of the tumor represented by different macro-molecules. Another characteristic is that each omic layer is characterized by tens of thousands of features like gene mutations [3] or gene expression. From a mathematical point of view tumors can be represented as vectors in a high dimensional space. This can be a problem in learning tasks known as the curse of dimensionality. This work focuses on the understanding of the available genomics data containing the somatic point mutations identified in each tumor sample. The availability of a large quantity of samples from the main tumor types and subtypes invites the study of current relations between different tumors and the development of learning algorithms that reduce the complexity of the initial high dimensional environment. The tumor samples are labeled by medical doctors and pathologists based on the tumor primary site and histology. The exploration of tumor mutational profiles can reveal communities of tumors and hidden relations between tumor types and subtypes [4]. This work aims to address the complexity of the pan-cancer somatic mutational data and learn a lower dimension of tumor representations based on the tumor mutational profiles.

At the same time of the significant growth in cancer biological data, the machine learning and deep learning communities have been developing learning methods such as Artificial Neural Networks with impressive results on image, signal and natural language processing [5]. One type of neural network model is the Auto-encoder (AE) [6]. AE are embeddings built to find reduced and simpler representations of complex data using un-supervised feedforward networks, therefore a non-linear reduction of dimensionality. Different types of Autoencoders have been proposed to generate a reduced latent space with a representative distribution of the original data using different regularization processes like Sparse [7] or contractive autoencoders [8].

The objective of this work is to learn a latent space of reduced dimensionality with autoencoders using mutational data from 14 types of tumors available from the International Cancer Genome Consortium (ICGC) [1] repository. This will allow understanding the similarities between tumors of different types and an improved classification performance of subtypes based on their mutational profile and their corresponding projection in a low dimensional latent space. The Kernel Target Alignment (KTA) score [9] and hierarchical clustering are proposed to measure the quality of the latent space. KTA is computed to measure the similarity between two kernel functions, one learned from the samples projected in the latent space and the second from an ideal target kernel. Kernel functions also lead to the measurement of similarities between training and test samples of the same class once the autoencoder model is trained and observe if the latent space maps similarly independent samples.

### Related work

Autoencoders have been used on a wide range of applications in cancer informatics. One application is its use on a single cancer type, such as liver cancer, while combining multi-omics data [10] to learn a latent space and identify new cancer subtypes. A similar case has been proposed for breast cancer to discover subtypes using transcriptomics data [11]. A newer version of AE, the Variational Auto-encoder, has been used to learn a latent space to improve the classification of known subtypes of lung cancer using DNA methylation data [12]. Moreover, instead of learning a latent space from a single type of cancer, a pan-cancer study based on transcriptomics data from The Cancer Genome Atlas (TCGA) [2] using Variational Auto-encoders evidenced a big potential for the use of autoencoders to learn reduced latent space while keeping biological insights [13]. Another work with gene expression data from TCGA applied standard autoencoders and Gene Supersets, which are a priori defined gene sets that retain biological signals in the latent space [14]. On the other hand, a network and graph theory analysis has been done for pan-cancer mutational data to detect communities of tumors [15] and find hidden relations between them using the co-occurrence of mutations as connections. A recent work maps mutated genes instead of the tumor samples to a lower dimension using deep learning techniques to learn a distributed representation [16]. By reviewing the bibliography, it is clear that data from different omics layers require models to simplify the original context and reflect emerging patterns. Autoencoders have shown great adaptability to biological data and are extremely useful for reducing dimensionality.

Our work proposes to learn a latent space from somatic mutations of large pan-cancer data using Autoencoders. This embedding is based in a model that projects tumor somatic mutation profiles in a low dimensional latent space where biological signals like tumor subtype persist and facilitates the comparison of tumor samples. For instance, this latent space can be used to explore mutational profiles when the primary tumor is unknown and there is no information on the tumor type, or to better classify tumor subtypes. From our best knowledge up to now, there are no attempts of reproducing a latent space using autoencoders from tumor somatic mutation data. Another important contribution of our work is an accurate tumor classification approach based on one-class Support Vector Machines (SVM) for each of the 40 tumor subtypes.

## Results

**Fig. 1:**
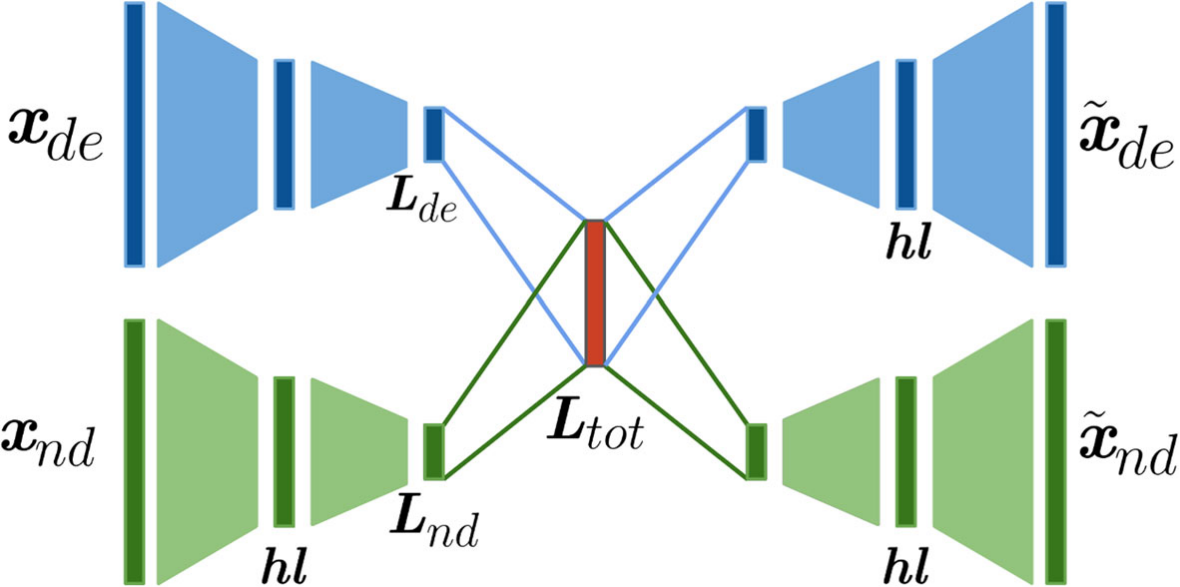
Model architecture. Scheme of the multi-modal autoencoder architecture for both deleterious and non-deleterious mutational profiles. Input and output dimension have 12424 genes. The encoder and decoder functions contain one hidden layer each of 400 activation functions (neurons). The latent layer of each autoencoder has 50 activation functions. Highlighted in red is the latent space *L*_*tot*_ which contains signal from both types of mutational profiles

In this work a neural network maps tumors characterized by mutational profiles from a high dimensional space, built from somatic mutated genes, to a low dimensional space using an Autoencoder as a nonlinear function. The mutational input data, which is highly sparse is considered as multi-modal since it is divided between deleterious and non-deleterious based on the variant type (see Fig. 1). The input tumor mutational profiles are transformed into a latent space as dense vectors.

**Fig. 2:**
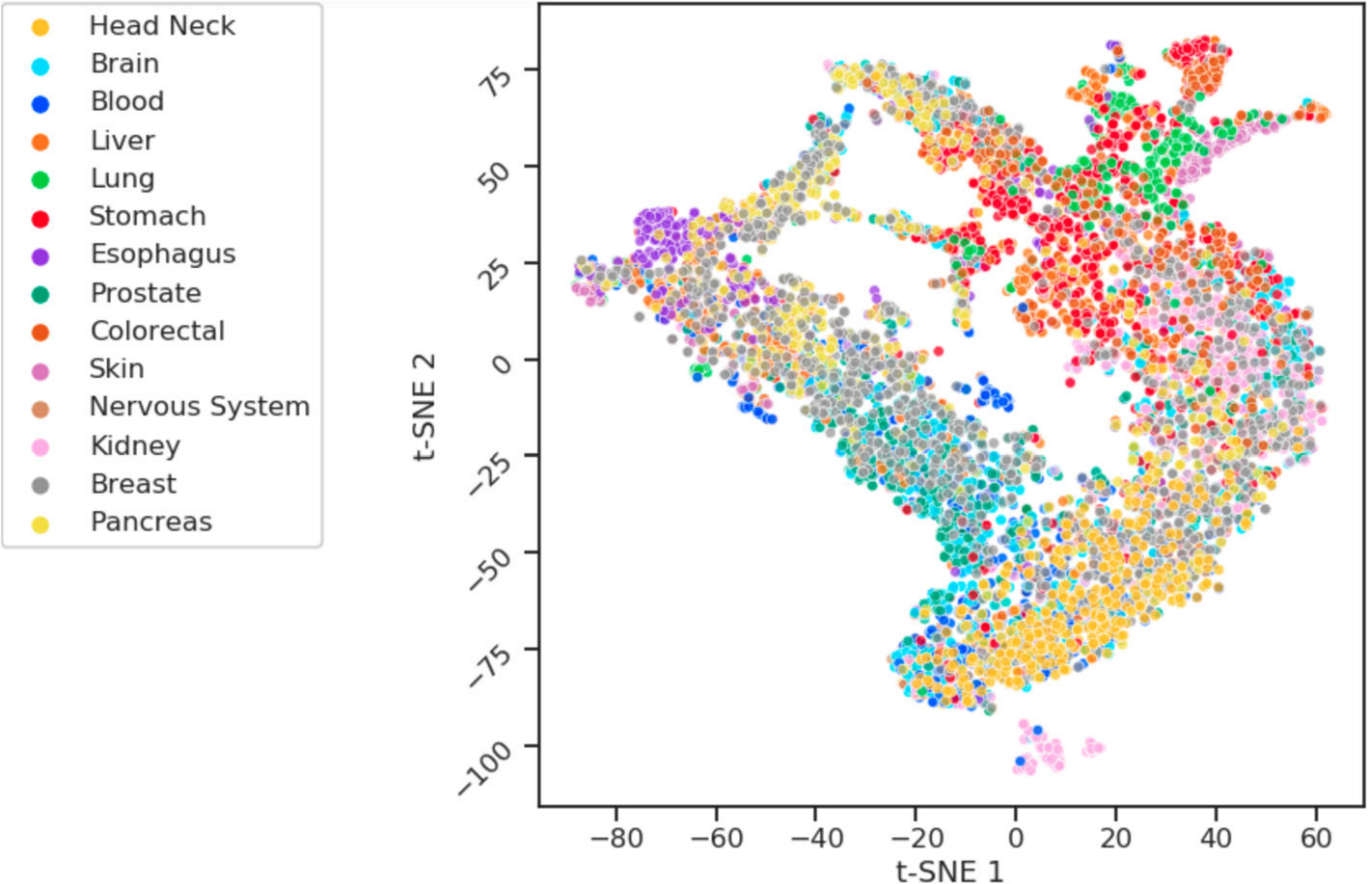
t-SNE scatter plot. Scatter plot of the projection of the latent space using t-SNE dimensions showing by different colors the 14 tumor types by primary site

By training a regularized autoencoder the tumors characterized with 12424 mutated gene as features are mapped to a final latent space of 50 dimensions. Thus, a global compression ratio of 248 is obtained. The learned latent space not only preserves the structural relationship between tumor subtypes but also improves the separability of classes making much easier the identification of a specific tumor phenotype. The resulting Autoencoder architecture has a multi-modal approach with one Encoder-Decoder function for deleterious and non-deleterious input mutations respectively. This allows weighting both types of input vectors (see “Methods” section). Then both models are merged at their respective latent layer level into a single global latent layer known as Latent Space. For regularization Batch Normalization is implemented after the Encoding hidden layer. Also, L2 norm is imposed to all the encoding weights to regularize their activity and penalize large weights. The learning rate and the L2 norm have been tuned by 5-fold cross validation using the validation loss computed as binary cross entropy. In the resulting latent space 40 one-class SVM models are trained, one for each tumor subtype. Then all the models are evaluated with independent tumor samples from Test set showing promising classification results. Figure 2 shows a scatter plot of a t-distributed stochastic neighbor embedding (t-SNE) as a projection of the resulting latent space after model training and evaluation [17].

### Quality assessment of latent space

**Fig. 3:**
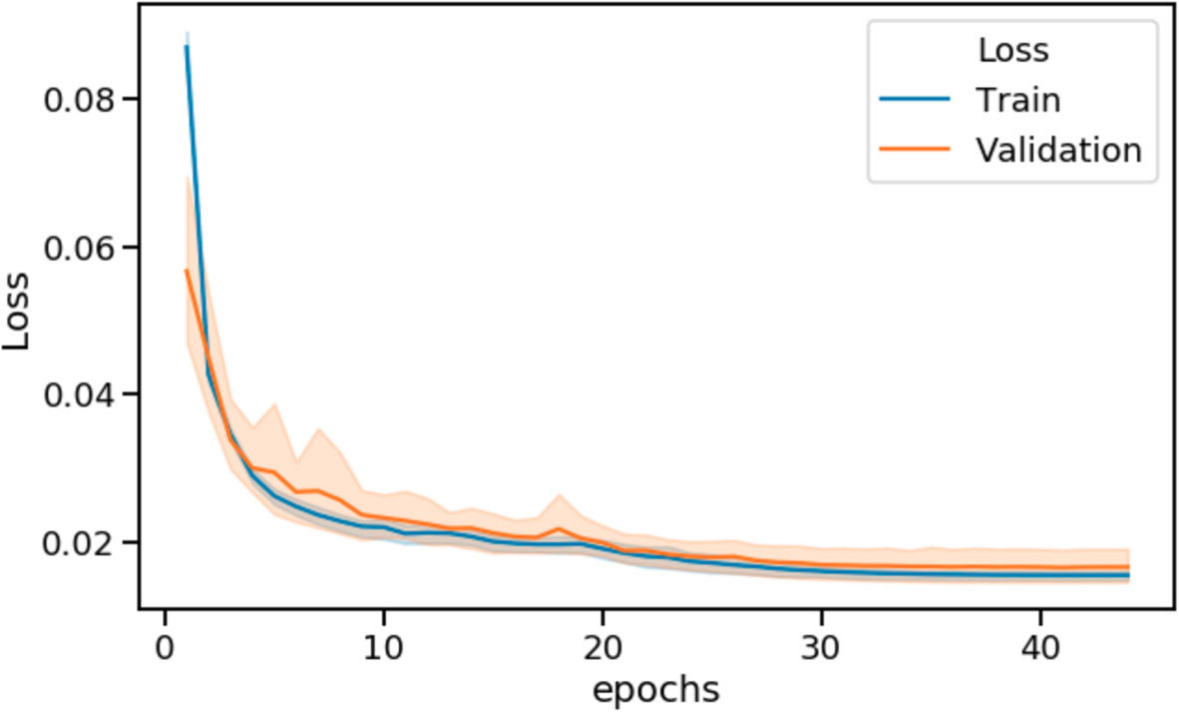
Validation loss. Autoencoder training and validation loss during training epochs after cross validation

The validation of the latent space must consider a set of quality assessments. In this work three different approaches are proposed. The first one is the reconstruction error of the autoencoder. Figure 3 shows the convergence of both the Training and Validation loss up to 45 epochs after 5 fold cross validation. This convergence means that the reconstruction quality stabilize. It serves as a way to measure how information is preserved from the input to the latent space until the output of the autoencoder. If the autoencoder loss is small means the reconstruction $\hat {x}$ is similar to the input *x* then the compressed latent vector preserves the salient features of the input space.

A second approach to assess the quality of the latent space is via Kernel Target Alignment. The KTA measures the kernel performance in finding high similarity between tumors of the same type, and low similarity between tumors of different types. The higher the KTA, the better the similarity between tumors of the same type. Given a gaussian kernel built in the latent space *K*_*l*_, a second kernel in the original input space *K*_*in*_, and the tumor type labels *Y*_*ty*_, the resulting alignment *KTA*_*l*_ obtained in the latent space outperforms the *KTA*_*in*_ obtained from the initial input space. The obtained results show that the autoencoder keeps the original input properties in the latent space while cleaning the noise, making a better space for pattern recognition tasks.

**Fig. 4:**
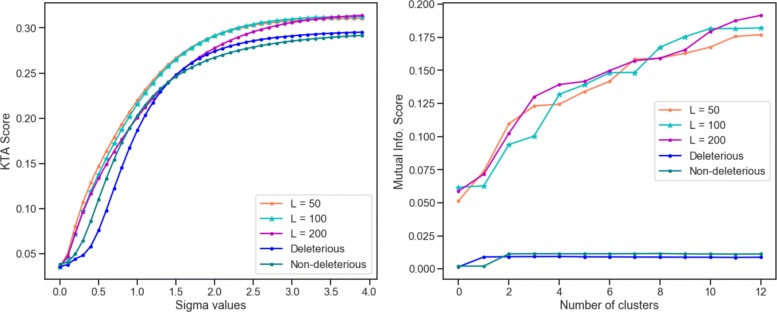
Latent Space evaluation. Left: Kernel target alignment score for different values of sigma parameter. Right: Mutual Information score for different number of clusters

Finally, the latent space is evaluated by cluster analysis. By performing hierarchical clustering in the input and in latent space separately, is possible to asses the quality of the resulting clusters by measuring how well tumors of the same type are clustered together. This is done by computing the mutual information score MI. This score consider the probability of a set of samples belonging to a class to be clustered together given a number of *k* clusters. As expected, the MI scores are better in the latent space when compared to the original input space. Figure 4 shows the results of KTA evaluation for different values of sigma parameter and the MI scores for different number of clusters. In order to evaluate different architectures of the autoencoder, other dimensions *L* of the latent space were evaluated, *L*=100 and *L*=200. As the assessment for different values of *L* leads to similar results, the *L*=50 is used in the final architecture since it has associated less model parameters or weights to fit during training and means a simpler model.

### Tumor sub-type classification

One-class SVMs is used to test if the low dimensional latent space learned captures mutational signals from the original input space and improves the tumor sub-types classification. A one-class SVM classifier is built for each of the 40 tumor subtypes. Every one-class classifier is trained with the samples corresponding to its subtype label and validated with the rest of the training samples. Finally, the one-class model is tested with an independent test set of samples preserved for autoencoder evaluation. The area under the ROC curve (AUC-ROC) is computed using the test samples to assess how well the classifier detects the True Positive samples, which in this case means tumors of the same subtype, therefore a correct classification. The same classification approach is also applied on the input space in both deleterious and non deleterious mutational data as a method to benchmark the obtained results. Table 1 shows the classification performance measured by the area under the ROC curve for each class on the latent space and the two input spaces *X*_*de*_ and *X*_*nd*_. Results equal to 0.5 indicates that the classifier can not find any difference between one subtype and the rest of the samples. On the other hand, results close to 1 correspond to classifiers that separates well the corresponding subtype class from the rest of the samples. The classification performance presents an improvement in the latent space in 35 out of 40 tumor subtypes (highlighted in the Table 1). In all these cases the area under the curve is higher in the classifier trained on the latent space than the ones trained on the input space with deleterious and non-deleterious mutational profiles. The tumor subtypes LAML-KR, KIRC-US, KIRP-US, LUSC-US, ESAD-UK, LIRI-JP and PRAD-CA show promising results with AUC-ROC close to 1 while the performance on the input space is close to 0.5.

**Table 1:** Classification results for 40 cancer subtypes

Primary site	Project name	Test samples	AUC latent	AUC De.	AUC Nd.
Head and neck	**ORCA-IN**	36	0.75	0.50	0.55
Brain	**LGG-US**	56	0.81	0.62	0.80
Blood	CLLE-ES	102	0.83	0.84	0.76
Head and neck	**THCA-SA**	28	0.82	0.66	0.81
Liver	**LINC-JP**	79	0.54	0.50	0.50
Lung	**LUSC-KR**	34	0.77	0.57	0.50
Skin	**GACA-CN**	24	0.79	0.50	0.50
Stomach	**LICA-FR**	49	0.67	0.50	0.51
Blood	**ESCA-CN**	65	0.77	0.5	0.61
Lung	**EOPC-DE**	40	0.81	0.74	0.55
Colorectal	**COCA-CN**	63	0.60	0.50	0.51
Skin	**SKCM-US**	67	0.84	0.50	0.50
Liver	**LICA-CN**	79	0.61	0.50	0.50
Blood	ALL-US	15	0.83	0.91	0.94
Skin	**SKCA-BR**	20	0.84	0.50	0.50
Brain	**GBM-US**	55	0.87	0.50	0.66
Nervous system	NBL-US	18	0.96	0.95	0.96
Blood	**LAML-KR**	41	0.97	0.73	0.64
Prostate	**PRAD-UK**	28	0.65	0.5	0.50
Prostate	**PRAD-US**	51	0.83	0.58	0.74
Blood	**MALY-DE**	49	0.82	0.50	0.50
Kidney	**KIRC-US**	82	0.90	0.50	0.58
Brain	**PBCA-DE**	90	0.79	0.77	0.59
Kidney	**RECA-EU**	49	0.84	0.5	0.5
Blood	AML-US	18	0.94	0.96	0.96
Breast	**BRCA-UK**	28	0.59	0.50	0.50
Kidney	**KIRP-US**	33	0.90	0.50	0.57
Prostate	**PRAD-CA**	58	0.88	0.71	0.50
Stomach	**STAD-US**	58	0.84	0.50	0.50
Stomach	**GACA-JP**	115	0.80	0.50	0.50
Liver	**LIRI-JP**	32	0.88	0.50	0.75
Breast	**BRCA-US**	189	0.75	0.60	0.50
Lung	**LUSC-US**	39	0.91	0.50	0.50
Esophagous	**ESAD-UK**	61	0.90	0.50	0.50
Colorectal	**COAD-US**	51	0.83	0.50	0.50
Breast	**BRCA-FR**	15	0.82	0.50	0.56
Pancreas	**PACA-AU**	73	0.72	0.60	0.50
Pancreas	PACA-CA	54	0.87	0.50	0.89
Head & Neck	THCA-US	76	0.85	0.85	0.50
Breast	**BRCA-EU**	114	0.79	0.56	0.50

The number of the test samples for the corresponding class is detailed. Area under the Roc curve is detailed for classifiers on Latent Space, Deleterious and Non-Deleterious input data. Tumor subtypes where the classification performance is improved in the latent space are highlighted in bold

## Discussion

Simple Somatic mutation data can be used to train an Autoencoder and build a latent space of lower dimensionality that keeps the biological signals of tumors. This study carries out a pan-cancer mapping by an Autoencoder trained with 8946 tumor samples from 40 tumor subtypes and evaluated with 2236 independent samples. The deleterious and non-deleterious variants in tumor mutational profiles are merged by a multi-modal autoencoder architecture allowing the weighting of each variant type differently. Although most pipelines for identification of disease-causing mutations filter out non-deleterious variants at the earliest stages, there is growing evidence that this type of variants affect protein splicing, expression and ultimately function, and some of these contribute to disease. This is not the case for tumor mutational profile exploration and classification, where non-deleterious variants showed to be very informative [4]. In this work deleterious and non-deleterious variant profiles equally contribute to the final latent space, with a mixture weight of *α*=0.5. It has been shown that Kernel Target Alignment and hierarchical clustering analysis exhibits an improvement on the latent space over these two input spaces regarding the capacity to group samples in clusters. Finally, a classification task using one-class approach is implemented in the latent space for each tumor subtype. The evaluation of the classifiers using independent samples for each class shows improvements in the vast majority of the tumor subtypes.

## Conclusions

This work presents a tumor mutation profile analysis pipeline which is from our best knowledge, the first attempt to learn a low dimensional latent space using autoencoders from mutational profiles of large pan-cancer tumor data. The latent space evidences biological signals in 50 dimensions after reducing the input dimension more than 200 times for 40 tumor subtypes. The use of kernel learning for latent space validation and assessment of the resulting cluster structures proved to be a useful approach. The use of a multi-modal approach to differentiate deleterious and non-deleterious variants let the autoencoder to learn a more realistic representation of the tumor somatic profiles. Classification at a tumor subtype level in the learned representation shows a clear improvement in comparison to the original input space. The quality of learned embedding has been assessed by different methods and proved to be a powerful tool for analysis of the pan-cancer mutational landscape.

This work is a first milestone and lay the foundations for future work on the learned somatic mutation autoencoder embedding to serve as a reference for biomarker discovery using feature selection techniques based on kernel learning and neural networks. If enough clinical data is available, the identified biomarkers with a further exploration of the latent space for cancer subtypes stratification could inform of patient expected prognosis and what are the most beneficial treatments. Future work should also consider further model validation and the inclusion of multi-omic input layers.

## Methods

### Pan-cancer somatic mutation data

Data has been downloaded from the International Cancer Genome Consortium [1]. Only Simple Somatic Mutation (SSM) data of the Release 27 has been considered for this work. Tumor data is labeled by type and subtype. There are 14 tumor types composed by 40 subtypes. There are a total of 11183 samples from whole exome sequecing and each one is characterized by more than 20.000 protein coding genes annotated with Variant Effect Predictor tool [18].

The pre-processing step consists of first counting separately the number of deleterious and non-deleterious somatic mutations per patient protein coding gene according to Cosmic notation [19]. Deleterious mutations are Inframe, Frameshift, Missense, Start Lost, Stop Gained and Stop Lost and the Non-deleterious are Synonimous, Splice, UTR 5 prime and UTR 3 prime. This results in two data matrices *X*_*mn*_ for each mutation type where *m* is the number of samples and *n* the number of genes or features. The value of each position *X*_*ij*_ corresponds to the number of somatic mutations a sample *i* has in gene *j*. Then each gene is zero-one normalized. It is important to remark the high sparsity of the data matrix and the curse of dimensionality. Initially the sample to feature ratio is 0.55. From this initial context only the features with non-zero values in at least 50 samples are retained and the rest that are only present in less than 50 samples are discarded. This decreases the feature set to a total of 12.424 genes and the resulting sample-to-feature ratio is 1.1 now. The data matrix is partitioned in train and test sets where train samples represent the 80% of the total data set. Within train set data where split in 5 folds to perform 5 training and validation iterations to tune the hyper-parameters of the model like learning rate, hidden layer size, regularization parameters, the number of training epochs and the mixture weight parameter.

### Autoencoders

Autoencoders are feedforward networks that learn two functions simultaneously: an encoder and decoder. The encoder maps the original input domain $\mathcal {X}$ to a new domain named latent space $\mathcal {Z}$ of dimension *L*. The decoder then maps from $\mathcal {Z}$ to the original input space $\mathcal {X}$. The mapping from $\mathcal {X}$ to $\mathcal {Z}$ is created by a neural network with one or multiple hidden layers [20]. The output of the decoder is also a reconstruction feedforward network. Since we aim to have a lower dimensionality at the latent space $\mathcal {Z}$, the autoencoder is forced to build an encoder function that captures all the salient features from the training data as much as possible [21]. The encoder and decoder functions are defined as ***z***=*f*(***x***) and $\tilde {\boldsymbol {x}} = g \left (\boldsymbol {z} \right)$ respectively where ***z*** are the samples at the learned latent space and $\tilde {\boldsymbol {x}}$ are the reconstructed samples on $\mathcal {X}$. With the previous definition, the autoencoder loss function to minimize is formalized as

$$E\left (\boldsymbol{x},\tilde{\boldsymbol{x}} \right) = E\left(\boldsymbol{x},g\left(f\left(\boldsymbol{x} \right) \right) \right) $$

where *E* penalizes *g*(*f*(***x***)) to be different to ***x***. In this work the measure of this loss function is the cross entropy score. Then the encoder *F* and decoder *G* functions can be defined as [22]

$$\begin{array}{*{20}l} \boldsymbol{z} = F\left(\boldsymbol{x}, \mathbf{W}_{F} \right) & = \sigma \left(\mathbf{W}_{F} \boldsymbol{x} + \mathbf{b}_{F} \right) \\ \tilde{\boldsymbol{x}} = G\left(\boldsymbol{z}, \mathbf{W}_{G} \right) & = \sigma \left(\mathbf{W}_{G} \mathbf{z} + \mathbf{b}_{G} \right) \end{array} $$

where *F*(·,**W**_*F*_) and *G*(·,**W**_*G*_) correspond to the encoding and decoding functions respectively and *σ*(·) is an activation function. The original input sample is $\textbf {x} \in \mathcal {X}$, $\tilde {\boldsymbol {x}} \in \mathcal {X}$ is the reconstructed samples and ***z*** the corresponding latent ones which dimension is lower than ***x***. The tensors **W** and **b** corresponds to the trained weights and biases of the encoder and decoder networks. These parameters are learned by backpropagation in order to minimize the loss function by the optimizer. This work uses Adaptive Moment Estimation (Adam) [23] optimizer to learn the weights of the network that minimizes the loss function. Adam is a novel first-order stochastic optimization technique. It computes an adaptive learning rate depending on the gradient mean.

Training an autoencoder to solely make $\tilde {\boldsymbol {x}}$ a copy of ***x*** does not ensure the learned latent space **z** is representative of the input ***x***. Without any constrain or penalization term, the encoder and decoder functions can result into a function that only copies the input in an output, but that is not useful to learn a latent space. For that reason different regularization strategies are evaluated which are L2 norm and Batch Normalization. L2 norm consists in a constraint term added to the loss function *E* where *β* is the regularization parameter.

$${E}'\left (\boldsymbol{x},g\left(f\left(\boldsymbol{x}\right)\right), \beta\left(f\left(\boldsymbol{x} \right) \right)\right) = E\left (\boldsymbol{x},g\left (f\left (\boldsymbol{x} \right) \right) \right) + \beta\sum_{i}\left | w_{i} \right |_{2}^{2} $$

The regularization term penalizes the functions *f* and *g* to have large weights leading to a simpler model and reducing overfitting [24]. To improve even more the generalization capacity the other regularization policy is used during the encoding process just after the first hidden layer of the encoding function. Batch Normalization [25] consists in auto-scaling the activation units to zero mean and unit variance at each mini-batch iteration.

Since the input data is characterized by two mutational data types and is represented in two matrices *X*_*de*_ and *X*_*nd*_ corresponding to deleterious and non-deleterious mutations respectively, the Autoencoder model must have two inputs and two outputs. Then, a multi-modal approach is proposed on the autoencoder architecture [26, 27]. A multi-modal autoencoder consists of two input networks and two output networks, each one with one Encoder and Decoder function. The network layers *L*_*de*_ and *L*_*nd*_ correspond to the latent representation of each model and are merged into one *L*_*tot*_ after the encoding function. This latent representation, which includes the signals of the two models, it is decomposed in two decoding functions. Figure 1 shows the proposed architecture. Since two models are participating in the construction of the final latent space, the final loss function is determined as follows

$$E_{tot} = \alpha E_{de} + (1-\alpha) E_{nd} $$

where *α* is a mixture weight parameter that represents the contribution of each model in the final latent representation, *E*_*de*_ is the loss of the deleterious model and *E*_*nd*_ is the non-deleterious. This approach allows to implement a weighting system on the input data and gives relative importance to deleterious and non deleterious mutational data. The best value of the *α* mixture weight parameter was found by a grid search of values *α*=[0.1,0.3,0.5,0.7,0.9], using Mutual Information (MI) from clustering results to evaluate the performance. During the cross validation task for each weight configuration a latent space is obtained, and based on the 14 tumor type classes a hierarchical clustering model with 14 clusters is implemented. For each clustering result the mutual information is calculated between the obtained cluster labels and the ground truth tumor labels. The final *α* parameter corresponds to the highest MI score obtained after cross validation which is *α*=0.5 (Additional file 1: Figure S1).

For the experiments, the architecture used consists in one hidden layer of 400 neurons (activation units) in both the encoding and decoding functions, named as *hl*_*e*_ and *hl*_*d*_ respectively. The latent space is obtained from the latent layer ***z*** with dimensionality *L* and represents an information bottleneck with the lowest dimension within the network. Different dimensions of latent space are evaluated to observe how the structure of the tumor data changes and is retained for each latent dimensionality. The pipeline’s objective is to reveal biological structures of the input data while reducing the dimensionality as much as possible. Figure 1 shows the proposed multi-modal architecture of the auto-encoder trained with both deleterious and non deleterious somatic mutational data from tumors.

During training the L2 norm and learning rate have been selected by 5-fold cross-validation on the train set using *L*_2_=[ 0.00005,0.00002,0.00001 ] and *L*_*r*_=[ 0.005,0.0025,0.001 ]. The final values are *L*_2_=0.00002 and *L*_*r*_=0.001. The number of epochs and the learning rate have been determined by an early stopping policy when the validation loss changes to lower than a certain threshold between each epoch.

### Kernel learning

In this work Kernel Learning is used to measure the structure of the learned latent space by the autoencoder and as the function used for the support vector classification step. Kernel functions can be thought as similarity functions between vectors. These functions indicate the dot product between those vectors mapped in a high dimensional Hilbert feature space. A Kernel is a function $k: \mathcal {X}\times \mathcal {X} \mapsto R$ where $\mathcal {X} \subseteq R^{n}$ is an n-dimensional space $\mathcal {X}$. The function *k* is symmetric and describes implicitly the mapping *ϕ* from $\mathcal {X}$ to a Reproducing Kernel Hilbert Space $\mathcal {H}$ by an inner product [28] $K\left (x_{i}, x_{j} \right) = \left \langle \phi (x_{i}), \phi (x_{j}) \right \rangle _{\mathcal {H}}$. The mapping from $\mathcal {X}$ to a feature space $\mathcal {H}$ is done by the function $\phi :X \mapsto \phi \left (X \right) \in \mathcal {H}$.

In this work, a good kernel finds high similarity between tumors of the same type and low similarity between tumors of different types. The kernel used is the Gaussian Kernel where the *σ* parameter functions as an exponential scaling factor.

1 $$ k(x_{i}, x_{j}) = \textup{exp}\left (- \frac{\left \| x_{i}-x_{j} \right \|^{2}}{2\sigma^{2}} \right); \sigma > 0  $$

The Gaussian kernel is one of the most common kernel functions. The parameter *σ* controls the size of the neighborhood of any *x*_*i*_ such that *k*(*x*_*i*_,*x*) is significantly larger than zero. The bigger the *σ* parameter, the more constant the function and thus the lower its ability to learn non-trivial patterns. On the other hand, low values of *σ* allow the kernel to fit complex patterns and be more sensitive to details [29].

Once the kernel is defined, it can be compared with other kernels via the Kernel Alignment [9]. Given two valid kernels *K*_1_ and *K*_2_ over a set of samples *M*, the alignment *A* between both kernels is defined as

2 $$ \mathit{A}\left (K_{1}, K_{2} \right) = \frac{\left \langle K_{1}, K_{2} \right \rangle_{F} }{\sqrt{\left \langle K_{1}, K_{1} \right \rangle_{F} \left \langle K_{2}, K_{2} \right \rangle_{F}}}  $$

and means the similarity between the two kernels using the same sample set *M* where 〈·,·〉_*F*_ is the Frobenius inner product between both kernel matrices. In other words, it can be thought as how similar both kernels map the samples. Considering the set *S* of labeled samples such that *S*={(*x*_1_,*y*_1_)...,(*x*_*m*_,*y*_*m*_)} where *x*_*i*_∈*R*^*n*^ and *y*_*i*_∈{−1,+1}, when *K*_2_=*yy*^*T*^ represents an ideal Kernel matrix or target *K*_*yy*_ with each position *K*_*ij*_=1 if *y*_*i*_=*y*_*j*_ and *K*_*ij*_=−1 if *y*_*i*_≠*y*_*j*_. The alignment of a kernel *K* and the target *K*_*yy*_ is known as the Kernel Target Alignment. The higher the KTA score, the bigger the inter-class distance, therefore the classes are more separated between each other and thus, well mapped to their corresponding target label. The *σ* parameter of the Gaussian Kernel has been tuned to maximize the corresponding KTA. In this work KTA is used to assess the quality of the latent space by using the tumor type labels and to evaluate the improvement of it in comparison with the original input space. It is expected to observe a higher KTA in the latent space and a lower one in the input high dimensional space.

Once the autoencoder is trained and the latent dimensions are finally defined, Kernel Alignment and support vector classification are used for latent space evaluation. It is important to remark that since there are 40 tumor subtypes, a one vs all approach using a binary classification is not the best option since classes are highly unbalanced. For this reason classification is done by the one class *ν*-SVM model [30]. It is used to classify each tumor sub-type against the rest of the tumor samples and is commonly used to define a decision boundary of only one class versus the rest of the sample set. This approach is applied to each tumor subtype and serves as a way to perform multi-class classification, where a one-class model is used instead of using a binary classifier. Its objective function is

$$\begin{array}{*{20}l} \underset{w,\xi,\rho }{\mathbf{min}} & \frac{1}{2}\left \| w \right \|^{2}+\frac{1}{n \nu}\sum_{i=1}^{n}\xi_{i} -\rho \\ \text{s.t.} & (w \cdot \phi (x_{i}))\geq \rho -\xi_{i}, \xi_{i}\geq 0 \\ \end{array} $$

The hyperparameter *ν*∈(0,1) functions as a lower bound on the number of samples characterized as support vectors and an upper one for the miss-classified samples that lie on the wrong side of the hyperplane. A set of slack variables *ξ*=(*ξ*_1_,...,*ξ*_*m*_) are introduced to allow the possibility of miss classifications when a sample fall on the wrong side of the margin. Then the decision function is defined as follows

$$f(x) = \textit{\textbf{sgn}}((w \cdot \phi (x_{i})) - \rho) $$

Note that a Kernel function can shape the decision function by the participation of the *ϕ*(·). The *f*(*x*) function will be positive for most of the samples in the training set in a small region which are going to be samples of the same tumor subtype, and -1 elsewhere. The closer *ν* parameter to 0 the penalization of miss-classified samples increases. If the training samples are separable from the origin, then the *ν*-SVM model will find a unique hyperplane that separates all the samples from the origin, and the distance from it to the origin is the maximal.

Once the classifiers are trained on the latent space these are evaluated with independent test samples. Classification performance is reported on Table 1.

### Cluster analysis

Latent space quality assessment is done also by cluster analysis as a complement of the KTA. Once the autoencoder network is trained, tumors are mapped from the original high dimensional space to a latent space $\mathcal {Z}$ with lower dimensionality. Given a latent space of dimension *L* and the original input space *X*_*de*_ and *X*_*nd*_, Hierarchical Clustering with a *k* number of clusters is applied separately to samples in $\mathcal {Z}$ on one side and to *X*_*de*_ and *X*_*nd*_ on the other. Clusters labels *c*_*i*_ are assigned to each sample *i* belonging to cluster *c*. Then by considering the real tumor types labels *Y*_*ty*_ as the ground truth, a mutual information score [31] is computed for each value of *k* to evaluate the quality of the obtained clusters in both cases. Every time the clustering algorithm is executed a set of *k* cluster labels *Λ*={*λ*_1_,...,*λ*_*k*_} and a set of ground truth labels *Y*={*y*_1_,...,*y*_*j*_} are defined. Then the mutual information score is defined as follows

$$\textup{MI}(\Lambda, C) = \sum_{k}\sum_{j}P\left (\lambda_{k} \cap y_{j} \right) \textup{log} \frac{P\left(\lambda_{k} \cap y_{j} \right)}{P(\lambda_{k})P(y_{j})} $$

where *P*(*λ*_*k*_) is the probability of a sample to be located in cluster *λ*_*k*_, *P*(*y*_*j*_) the probability to belong to class *y*_*j*_ and *P*(*λ*_*k*_∩*y*_*j*_) the one to be at the intersection of both. The possible results of the score are MI∈(0,1). The higher the MI score the better the quality of the obtained cluster result. In this work, the MI score is computed for different values of clusters *k* in both the obtained latent space $\mathcal {Z}$ and the original input space *X*_*in*_. If the quality of clusters is better in the latent space than the input space then MI_*z*_ will be higher than MI_*de*_ and MI_*nd*_. A higher value of MI can be interpreted as samples of the same tumor type tend to be grouped together in the same cluster.

### Computational tools

Data preprocessing, clustering analysis and kernel learning have been implemented with Python 3.6 and Scikit Learn [32]. The autoencoder model has been built with Keras an Tensorflow backend. Training has been performed with a GPU N-Vidia GTX 1060 4GB.

## Supplementary information

**Additional file 1** Supplementary Figure S1.

## Data Availability

The data that support the findings of this study are available from the International Cancer Genome Consortium (ICGC) but restrictions apply to the availability of these data, which were used under license for the current study, and so are not publicly available. Data are however available from the authors upon reasonable request and with permission of ICGC.

## Abbreviations

AE: Autoencoders
AUC-ROC: Area under the ROC curve
ICGC: International cancer genome consortium
KTA: Kernel target alignment
MI: Mutual information
SSM: Simple somatic mutation
SVM: Support vector machines
TCGA: The cancer genome atlas
t-SNE: t-distributed stochastic neighbor embedding
