# Supplementary material for: A pan-cancer somatic mutation embedding using autoencoders
Source: BMC Bioinformatics. 2019 Dec 11;20:655. doi: 10.1186/s12859-019-3298-z (PMC6907172; doi:10.1186/s12859-019-3298-z)
Supplement: Supplementary file 1 — Additional file 1 Supplementary Figure S1. [file 12859_2019_3298_MOESM1_ESM.pdf]

Supplementary material:

A Pan-cancer Somatic Mutation Embedding using Autoencoders

Martin Palazzo[1,2,3], Pierre Beauseroy[2] and Patricio Yankilevich[1*]

Author affiliation:

[1] Instituto de Investigación en Biomedicina de Buenos Aires (IBioBA) - CONICET - Partner Institute of the Max Planck Society, Buenos Aires, Argentina

[2] Institut Charles Delaunay, Universite de Technologie de Troyes, Troyes, France

[3] Universidad Tecnologica Nacional, FacultadRegional Buenos Aires, Argentina

[*] Corresponding author

Correspondence:

Patricio Yankilevich   Email: pyankilevich@ibioba-mpsp-conicet.gov.ar

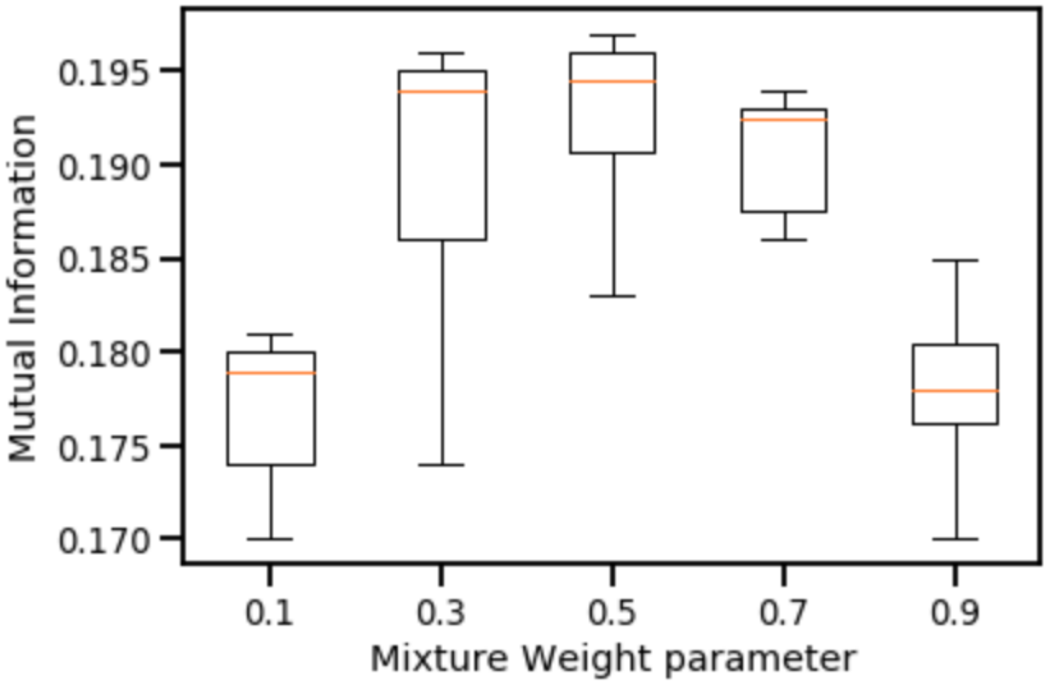

Figure S1. Mixture weight parameter. Mutual information values after cross validation for different values of α parameter. The final value of the mixture weight parameter is α = 0.5 since it maximizes the mutual information score.
